# Influence of Laser Marking Parameters on Color Generation in AISI 304 Stainless Steel

**DOI:** 10.3390/ma19030612

**Published:** 2026-02-05

**Authors:** Lyubomir Lazov, Nikolay Angelov, Jurijs Dehtjars, Edmunds Sprudzs, Arturs Abolins, Petar Tsvyatkov

**Affiliations:** 1 Rezekne Academy, Riga Technical University, LV-1658 Riga, Latvia; angelov_np@abv.bg (N.A.); jurijs.dehtjars@rtu.lv (J.D.); edmunds.sprudzs@rtu.lv (E.S.); arturs.abolins_1@rtu.lv (A.A.); 2 Department of Machine and Precision Engineering, Technical University of Gabrovo, 5300 Gabrovo, Bulgaria; ppeter@mail.bg

**Keywords:** laser marking, AISI 304 steel, raster step, speed, roughness, scanning overlap coefficient, color difference, surface morphology

## Abstract

The study concerns the influence of some basic parameters (speed, raster step, scan overlap coefficient, and surface modification at different processing angles) on the process of color laser marking with a fiber laser on AISI 304 stainless steel samples. Different surface morphology was obtained for single-shot marking; double-shot marking at angles 0° and 90°; and triple-shot marking at angles 0°, 60°, and 120°. According to the created methodology, dependencies for surface roughness, resulting color, color difference, and chromatic distance from the parameters raster step and scanning speed were established. The resulting colors and color differences for different values of these parameters for the three resulting morphological surfaces were compared. Trends in color saturation changes were established for single-shot, double-shot, and triple-shot color marking, as well as for changes in technological parameters in the studied intervals.

## 1. Introduction

Laser-based coloration of metallic surfaces, particularly stainless steel and titanium alloys, has gained significant attention due to its ability to generate stable, non-toxic, and corrosion-resistant colors without the need for pigments. This process, which modifies ultra-thin oxide films, is highly relevant in industries such as medical device manufacturing, consumer electronics, aerospace, and anti-counterfeiting technologies. Unlike traditional chemical methods, laser color marking offers a precise, localized, and digitally controlled approach, making it particularly effective for metals like stainless steel and titanium alloys [1,2,3].

The mechanisms underlying laser-induced coloration primarily involve interference coloration, which arises from changes in the oxide film thickness, and light scattering, which results from the formation of micro- and nano-scale surface structures during laser irradiation. These effects are influenced by various laser parameters, including pulse duration, fluence, scanning speed, and line overlap. Nanosecond lasers, in particular, are widely used for generating high-precision color patterns on stainless steel and titanium alloys due to their ability to deliver finely controlled pulses that modify the surface properties effectively [4,5].

The formation of oxide layers ranging from 20 to 350 nm in thickness enables the generation of a broad spectrum of visible colors, which depend on both the wavelength of light and the oxide layer thickness. Recent studies have not only explored the aesthetic potential of laser-induced coloration but also its use in anti-counterfeiting applications. By embedding unique microstructures within the oxide layer, these markings can serve as security features for product traceability [6,7]. The structural modifications not only enhance the appearance of the metal surface but also improve its functional properties, making this technique highly valuable in industries where durability, aesthetic appeal, and material integrity are critical [8].

One of the main challenges in laser-based coloration is optimizing laser parameters to achieve the desired color results. Parameters such as pulse duration, repetition frequency, fluence, and scanning speed directly influence both the thickness and composition of the oxide layer, as well as the surface morphology. Studies have shown that factors like surface melting, thermal plasma formation, and the creation of laser-induced periodic surface structures (LIPSSs) can significantly alter the specular and diffuse light reflection, thus affecting the perceived color under varying illumination conditions [9,10,11,12,13].

The laser scanning strategy also plays a crucial role in determining the final coloration. Common methods include single-direction scanning, which generates anisotropic grooves that affect directional optical scattering; cross-scanning at 90°, which improves color uniformity; and triple-direction scanning with 60° rotation, which creates quasi-isotropic textures, enhancing color saturation and minimizing viewing-angle dependence. Additionally, the overlap coefficient between adjacent raster paths influences surface roughness and oxide coloration, with higher overlap values resulting in smoother surfaces and more uniform colors [14,15,16,17].

A method is reported in paper [18] to erase selectively the oxide-based colors using laser-induced oxygen reduction. Especially, the color marks are reprocessed in a low-oxygen environment employing a nanosecond laser. A low fluence was used in order to diffuse oxygen out into the atmosphere and yield a lower form of metal oxides or a pure metal. Any cumulative fluence exceeding 25 J/cm^2^ was sufficient to erase any laser-induced colors on titanium substrates. The analysis revealed that all fields were mainly composed of TiO_2_ prior to erasing with only small contributions from Ti_2_O_3_ and TiO/TiN. Following the proposed laser-induced oxygen reduction, the relative concentration of TiO_2_ decreased substantially, while the overall amount of Ti in the near-surface region increased. The results clearly show that the erasing of oxide-based color marks is only due to oxygen diffusion back into the atmosphere, and there were not any signs of laser ablation.

Advances in scanning electron microscopy (SEM) and other surface characterization techniques have provided deeper insights into how laser parameters influence oxide layer formation, phase composition, and resulting color changes. These developments offer more precise control over the material’s surface properties and help clarify the mechanisms behind laser-induced microstructural modifications [19,20].

This research aims to explore the relationship between laser parameters, surface roughness, and scanning strategies in the nanosecond laser marking of AISI 304 stainless steel. By systematically varying parameters such as scanning speed, overlap coefficient, and scanning strategy, the study will evaluate their effects on oxide growth, surface roughness, and color generation. The results will offer valuable insights into optimizing laser color marking processes for industrial applications where precision and reproducibility are essential.

## 2. Material, Equipment, and Methods

### 2.1. Material: AISI 304 Stainless Steel

The material studied is stainless steel AISI 304 (ThyssenKrupp, Essen, Germany), and it is austenitic steel. It has excellent corrosion resistance, good mechanical properties, and low cost, which contribute to its widespread use in industry. It is used for making pipes, parts of furnace fittings, heat exchangers, muffles, retorts, nozzles, collectors of exhaust systems, electrodes of spark plugs, welding devices, and vessels of chemical engineering, operating at a temperature from –196 °C to 600 °C in environments of medium activity.

The chemical composition and some physical properties of the steel are presented in Table 1 and Table 2, respectively.

### 2.2. Laser System

A laser technology system with a fiber laser Rofin PowerLine F 20 Varia (Rofin-Baasel, Munich, Germany) was used to conduct the experiments. Some basic technical parameters of the laser system and the laser are presented in Table 3. The laser operates in the near-infrared range and is in pulsed mode. It is characterized by excellent beam quality, high frequency, and good efficiency. The laser system has high positioning accuracy and provides a wide range of scanning speed *v* variation.

### 2.3. Experimental Methodology

**Figure 1:**
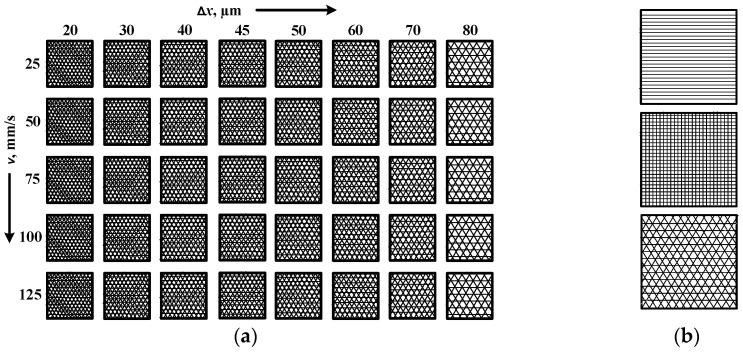
(**a**) Scheme of the matrix for conducting experiments for triple triple-pass marking. (**b**) Test area (square) for single-, double-, and triple-pass marking.

To study the influence of the laser parameters raster step and scanning speed on the roughness, color, and color difference of the obtained markings, three matrices for single-, double-, and triple-pass marking on the surface of the samples were designed. They are composed of 5 rows with 8 squares in each row (Figure 1a). The squares are marked in a raster manner, with each square being marked with a different raster step and speed. The scanning speed of each row is different and is in the range from 25 mm/s to 125 mm/s in increments of 25 mm/s. For each row, the raster pitch Δ*x* varies from 20 μm to 80 μm. The presented matrix is for three-pass raster marking (at angles 0°, 60°, and 120°). The other two matrices are identical as for two-pass marking the angles are 0° and 90°, and for single marking the angle is 0°. Figure 1b shows the individual test areas for single-, double-, and triple-pass marking with the corresponding laser beam paths plotted.

**Figure 2:**
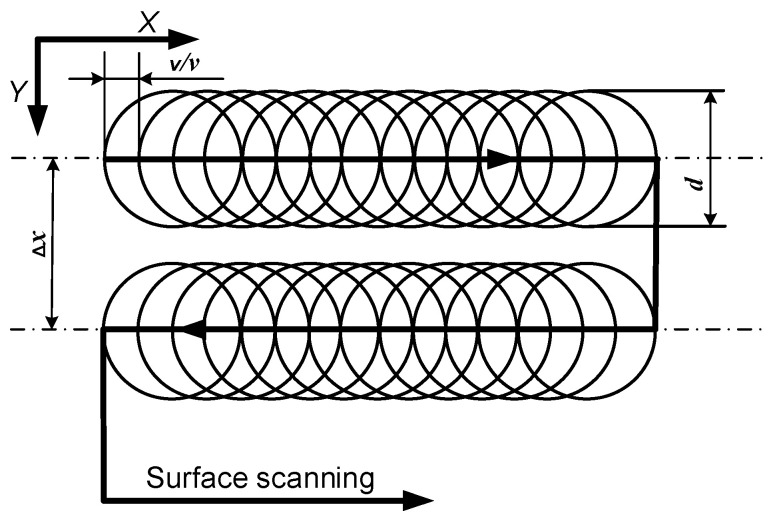
Scheme of raster laser marking by X and Y of the tested sample.

The degree of overlap in the X direction depends on the diameter *d* of the working spot, the frequency *v*, the scanning speed *v*, and the raster step Δ*x* (Figure 2). The pulse overlap coefficient *k_poc_* is given by the expression

*k_poc_* = [1 − *v*/(*ν* × *d*)] × 100% (1)

The influence of the pulse overlap coefficient *k_poc_* will not be discussed in the present study, since for the values of the specific parameters in the study, the frequency of 20 kHz, the diameter of the working spot *d* of 70 μm, and the scanning speed *v* in the interval from 25 mm/s to 125 mm/s, it has values from 91.1% to 98.2%. These values are suitable for laser color marking.

The degree of overlap in the Y direction depends on the diameter of the working spot and the raster step Δ*x*. The scanning overlap coefficient *k_soc_* is given by the expression

*k_soc_* = (1 − Δ*x*/*d*) × 100% (2)

In the studies, the raster step Δ*x* changes from 20 μm to 80 μm and changes in a very wide interval, even having a negative value for a raster step Δ*x* of 80 μm. This study discusses how the raster step Δ*x* (respectively the scanning overlap factor) affects surface roughness *R_a_*, resulting colors, and color differences.

### 2.4. Surface Roughness Measurements

The surface roughness of the marked areas was analyzed using the OLS5100 laser microscope for materials analysis, which provides 3D surface characterization with high resolution and nanometer precision. This instrument combines confocal laser scanning technology with advanced optical profiling algorithms, allowing for accurate measurement of both micro- and nano-scale surface features. The system captures detailed topographic data on selected areas and reconstructs the surface in three dimensions, allowing the extraction of key roughness parameters such as *R_a_*, *R_z_*, and *R_q_*.

Its vertical resolution in the nanometer range and precise autofocus system ensure high repeatability and low measurement uncertainty. Key specifications are given in Table 4.

Roughness measurements were performed at the center of each marked square area to avoid edge effects. Topographic scanning was performed on a fixed rectangular area chosen to be large enough to capture the characteristic surface texture. Based on the laser spot diameter (70 μm) and the raster steps used (20–80 μm), a standardized field of view was chosen to encompass several laser paths and the valleys between them. The typical measurement area for the presented results was about 250 μm × 250 μm.

To ensure statistical reliability and to account for possible local inhomogeneity, seven separate roughness measurements were performed, and the average value was calculated for each marked square.

This methodology provided a highly accurate, reproducible, and statistically valid quantitative assessment of surface roughness.

### 2.5. Color Characterization of Laser-Marked Surfaces

The color characteristics of the laser-marked areas were evaluated using digital image analysis. Images of the processed surfaces were acquired using a digital camera under controlled and repeatable illumination conditions. The camera was operated in manual mode with fixed exposure parameters, white balance, and focal distance to minimize variations caused by imaging conditions. Diffuse illumination was applied to reduce specular reflections from the metallic surface.

All images were imported into Adobe Photoshop 2024 (v25.x) (Adobe Systems Inc., San Jose, CA, USA) and converted to the sRGB IEC 61966-2-1 color space to ensure consistency across all samples. No automatic contrast enhancement, color correction, or filtering was applied. Homogeneous regions of the laser-colored areas were selected, avoiding scan edges, defects, and highly reflective zones. The RGB color coordinates were extracted using the Eyedropper Tool with an averaging window of 5 × 5 pixels. For each sample, measurements were performed at multiple locations, and the mean RGB values were calculated to improve statistical reliability.

To quantitatively determine the color contrast between the laser-marked surfaces and the untreated material, the color difference ΔE* was calculated. To ensure accuracy corresponding to human perception, the extracted RGB coordinates were converted from the sRGB color space to the CIE L*a*b* color space. The color difference ΔE* is defined as the Euclidean distance between the color coordinates of the marked area and those of the reference untreated surface, using the following equation:

(3) $${\Delta E}^{*}=\;\sqrt{{\left(L^{*}-\;L_{0}^{*}\right)}^{2}+\;{\left(a^{*}-\;a_{0}^{*}\right)}^{2}{+\;(b^{*}-\;b_{0}^{*})}^{2}}\;$$

L*a*b* are the color coordinates in the CIE L*a*b* space for the laser marking surface, and L_0_*a_0_*b_0_* are the color coordinates for the untreated surface of the AISI 304 steel (reference value).

The calculated value of ΔE* by formula (3) allows for a direct comparison of the degree of coloration obtained under different laser processing parameters and integrates changes in lightness, hue, and saturation into a single measure.

### 2.6. Investigated Functional Dependencies in the Conducted Experiments

To achieve the objective of evaluating how laser marking parameters influence roughness and the resulting color characteristics of AISI 304 stainless steel, the following specific research tasks were set:

- The role of the scan overlap coefficient *R_a_* = *R_a_* (*k_soc_*);
- The role of the raster step on the roughness *R_a_* = *R_a_* (Δ*x*) in single-, double-, and triple-pass marking;
- The role of the raster step Δ*x* on the resulting surface color in single-, double-, and triple-pass marking;
- The role of the speed *v* on the resulting surface color in single-, double-, and triple-pass marking;
- The role of the raster step Δ*x* on the color difference in single marking.

## 3. Results

### 3.1. Influence of Raster Step on Surface Roughness and RGB Color Values

#### 3.1.1. Single Repetition (N = 1) for Roughness

**Figure 3:**
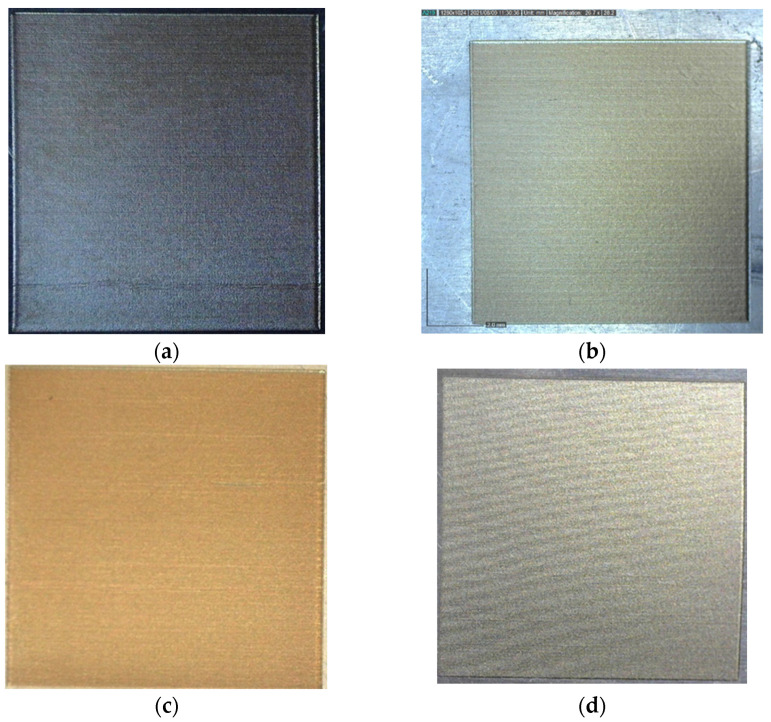
Photographs of laser-marked fields with the following parameters: (**a**) 20 μm and 25 mm/s, (**b**) 30 μm and 50 mm/s, (**c**) 20 μm and 75 mm/s, and (**d**) 40 μm and 125 mm/s.

For single repetition (*N* = 1), Figure 3 presents raster marked fields with a fiber laser on AISI 304 stainless steel samples with the following parameters: (a) 20 μm and 25 mm/s, (b) 30 μm and 50 mm/s, (c) 20 μm and 75 mm/s, and (d) 40 μm and 125 mm/s. In the first two cases, the laser marking process is realized by melting, and in the third and fourth cases, laser marking by oxidation is obtained.

**Figure 4:**
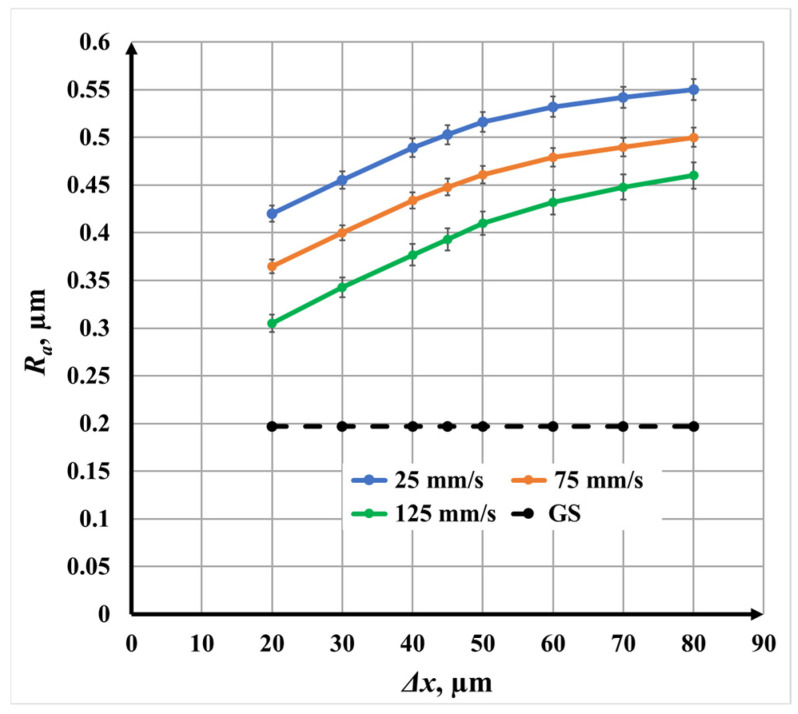
Graphics of the dependence of roughness on the raster step for single repetition for three *v* speeds: blue color—25 mm/s; orange color—75 mm/s; and green color—125 mm/s. The black dotted line is the roughness of the unprocessed surface.

The influence of raster step Δ*x* on surface roughness and color contrast during nanosecond fiber laser marking of AISI 304 stainless steel are analyzed (Figure 4). The analysis is performed separately for single, double, and triple scanning repetitions, followed by a comparative discussion. The raster step was varied within the interval of 20 μm to 80 μm, corresponding—according to Equation (2)—to a scanning overlap coefficient ranging from 85.7% to −14.3% for a focused laser spot diameter *d* of 70 μm. Three marking speeds *v* were investigated: 25 mm/s, 75 mm/s, and 125 mm/s. The initial roughness of the untreated surface was *R_a_* = 0.197 μm, while all other laser parameters were kept constant (Table 5).

Figure 3 presents the dependence of surface roughness *R_a_* on raster step Δ*x* for single-pass laser marking at the three investigated *v* scanning speeds. For all speeds, an increase in raster step leads to a nonlinear increase in surface roughness, indicating that the spatial distribution of laser energy plays a dominant role in surface morphology formation.

A distinct change in slope is observed in two raster step Δ*x* intervals:

- From 20 μm 50 μm, where the roughness *R_a_* increases rapidly;
- From 50 μm 80 μm, where the increase becomes more gradual.

This behavior correlates directly with the scanning overlap coefficient. In the first interval, the overlap remains high, leading to repeated irradiation of the same surface regions, enhanced thermal accumulation, and pronounced surface melting. In the second interval, the overlap decreases substantially, reducing energy accumulation and limiting further roughness growth.

The roughness variation intervals are as follows:

- At *v* = 25 mm/s, *R_a_* increases from 0.42 μm to 0.55 μm;
- At *v* = 75 mm/s, *R_a_* increases from 0.365 μm to 0.50 μm;
- At *v* = 125 mm/s, *R_a_* increases from 0.305 μm to 0.46 μm.

The calculated roughness growth rates confirm this trend. For Δ*x* ∈ [20, 50] μm, the rate of roughness increase is approximately three times higher than for Δ*x* ∈ [50, 80] μm for all speeds. Moreover, at identical raster steps Δ*x*, the roughness *R_a_* at *v* = 25 mm/s is approximately 14% higher than at *v* = 75 mm/s and 32% higher than at *v* = 125 mm/s, reflecting stronger thermal effects at lower scanning speeds.

#### 3.1.2. Color Characteristics R/G/B for Single-Pass Marking

The colorimetric results for single-pass marking are summarized in Table 6, where each marked square is identified by its scanning speed and raster step position. Most of the obtained colors fall within the yellow spectral region, with noticeable variations in saturation.

**Figure 5:**
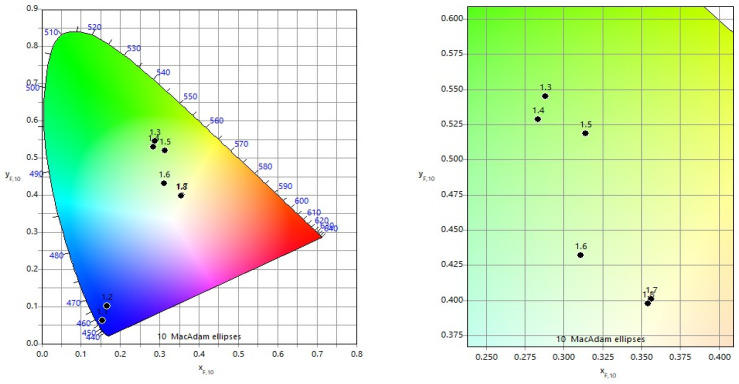
Color diagram for speed *v* = 25 mm/s and raster step Δ*x* in interval from 20 μm to 80 μm.

Figure 5 illustrate the color diagram for *v* = 25 mm/s. At small raster steps (20–30 μm), the combination of low speed and high overlap leads to intense energy absorption, partial melting, and the formation of darker colors, including blue tones. As the raster step increases, the overlap coefficient decreases, shifting the coloration mechanism toward oxidation-dominated processes. This results in a transition through green shades and finally to yellow at Δ*x* = 80 μm, where the overlap coefficient becomes negative.

**Figure 6:**
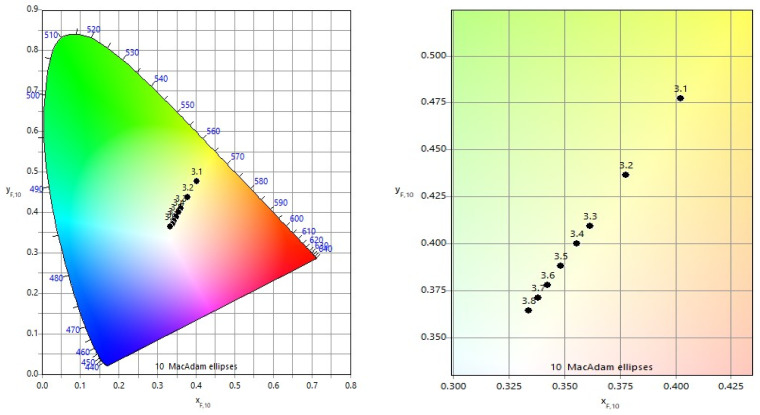
Color diagram for speed *v* = 75 mm/s and raster step Δ*x* in interval from 20 μm to 80 μm.

For *v* = 75 mm/s (Figure 6), the absorbed energy is significantly lower. All marked areas remain within the yellow region, but a clear decrease in color saturation is observed with increasing raster step. The darkest yellow corresponds to Δ*x* = 20 μm, while the lightest occurs at Δ*x* = 80 μm, reflecting progressively thinner oxide layers.

**Figure 7:**
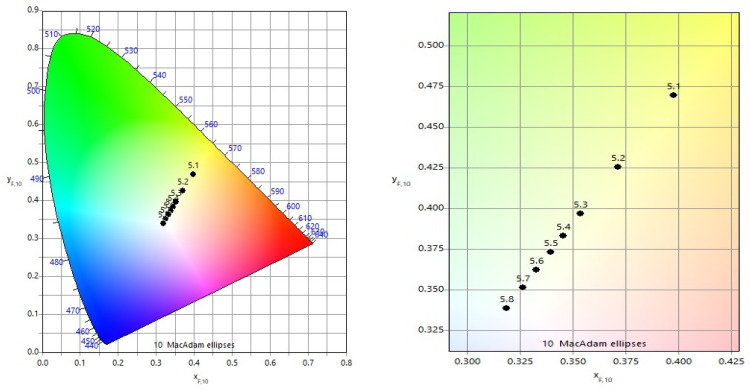
Color diagram for speed *v* = 125 mm/s and raster step Δ*x* in interval from 20 μm to 80 μm.

At *v* = 125 mm/s (Figure 7), the same trend persists, but the colors are generally less saturated than at *v* = 75 mm/s, confirming that higher scanning speeds reduce oxide growth and suppress intense interference coloration.

Overall, for single repetition, increasing raster step leads simultaneously to the following:

- Increased surface roughness;
- Reduced oxide layer thickness;
- Decreased color saturation.

#### 3.1.3. Double-Pass Marking (N = 2, Angle: 0°/90°) for Roughness

**Figure 8:**
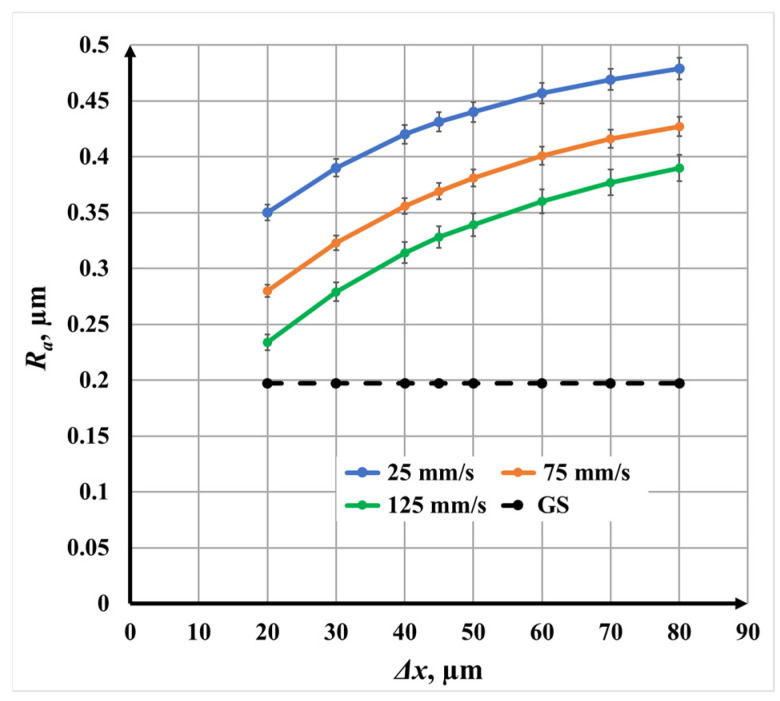
Graphics of the dependence of roughness on the raster step for double repetition for three speeds *v*: blue color—25 mm/s; orange color—75 mm/s; and green color—125 mm/s. The black dotted line is the roughness *R_a_* of the unprocessed surface.

For double-pass marking, the roughness dependence on raster step is shown in Figure 8. The overall trend remains nonlinear, with steeper roughness growth at smaller raster steps. However, the roughness values are consistently lower than those observed for single repetition.

Specifically,

- At *v* = 25 mm/s, *R_a_* varies from 0.35 μm to 0.48 μm;
- At *v* = 75 mm/s, from 0.28 μm to 0.43 μm;
- At *v* = 125 mm/s, from 0.23 μm to 0.39 μm.

At a fixed raster step, the roughness at *v* = 25 mm/s is approximately 20% higher than at *v* = 75 mm/s and 40% higher than at *v* = 125 mm/s. The reduction in roughness compared with single repetition is attributed to the cross-scanning strategy, which redistributes material and partially smooths anisotropic surface features.

#### 3.1.4. Triple-Pass Marking (N = 3, Angle: 0°/60°/120°) for Roughness

**Figure 9:**
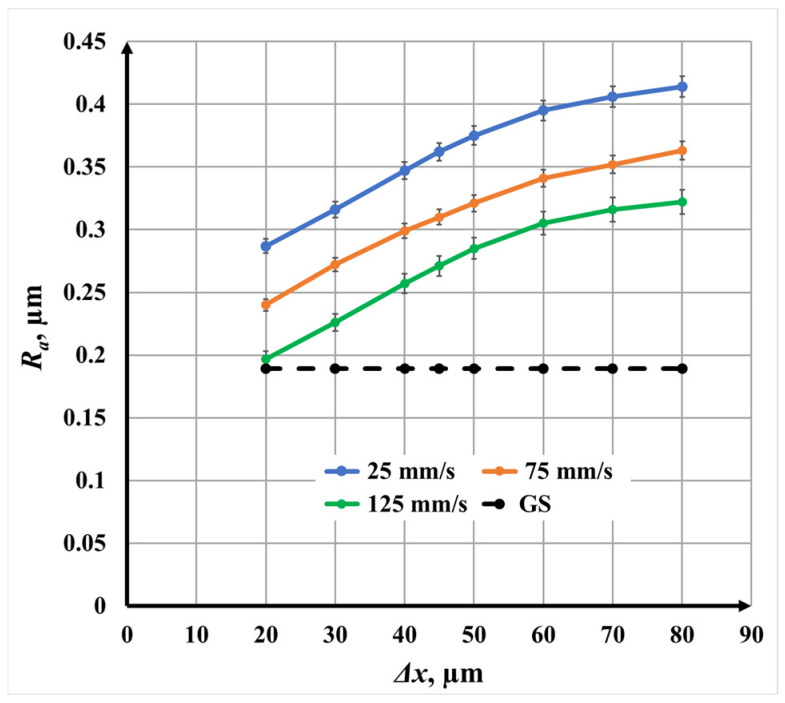
Graphics of the dependence of roughness on the raster step Δ*x* for triple-pass marking for three speeds at *v*: blue color—25 mm/s; orange color—75 mm/s; and green color—125 mm/s. The black dotted line is the roughness of the unprocessed surface.

Figure 9 presents the roughness *R_a_* dependence for triple-pass marking. The nonlinear trend with raster step is preserved; however, triple repetition produces the lowest roughness *R_a_* values among all investigated scanning strategies.

Measured values indicate the following:

- 0.29–0.41 μm at at *v* = 25 mm/s;
- 0.24–0.36 μm at at *v* = 75 mm/s;
- 0.20–0.32 μm at at *v* = 125 mm/s.

At equal raster steps, the roughness *R_a_* at *v* = 25 mm/s is approximately 17% higher than at *v* = 75 mm/s and 37% higher than at *v* = 125 mm/s. The quasi-isotropic energy distribution achieved by triple scanning promotes surface homogenization and suppresses excessive roughness *R_a_* development.

#### 3.1.5. Comparative Analysis at *v* = 25 mm/s for Roughness

**Figure 10:**
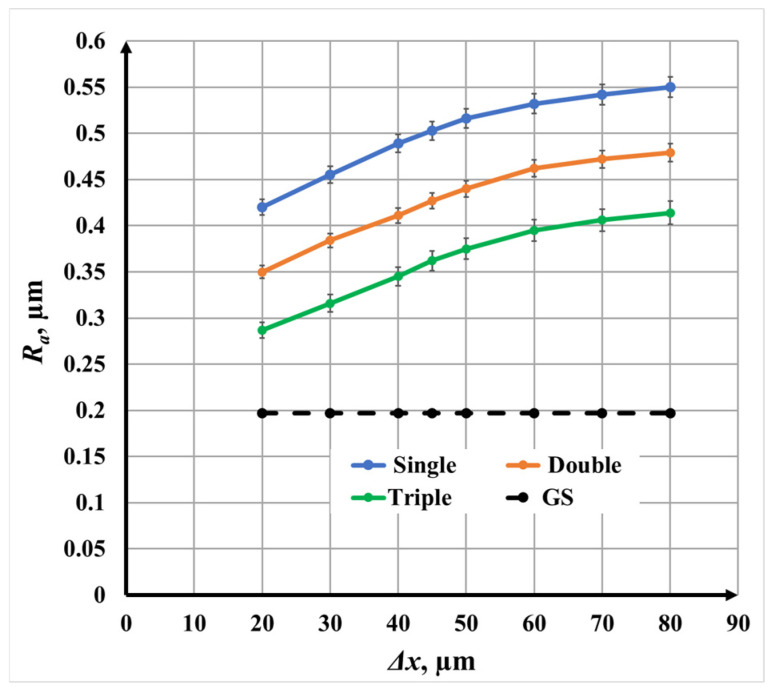
Graphics of the dependence of roughness *R_a_* on the raster step for speeds *v* = 25 mm/s: blue color—single repetition; orange color—double repetition; and green color—triple repetition. The black dotted line is the roughness of the unprocessed surface.

A direct comparison of single-, double-, and triple-pass marking at *v* = 25 mm/s is presented in Figure 10. For all scanning strategies, increasing raster step Δ*x* results in a nonlinear roughness *R_a_* increase. However, the absolute roughness levels differ significantly:

- Single-pass marking produces the highest roughness *R_a_*;
- Double-pass marking reduces roughness *R_a_* by approximately 17%;
- Triple-pass marking reduces roughness *R_a_* by approximately 50% relative to single marking.

This comparison demonstrates that scanning strategy is as influential as raster step Δ*x* in controlling surface roughness *R_a_*. Multi-directional scanning not only modifies surface morphology but also enhances oxide uniformity, which is critical for stable and reproducible color generation.

### 3.2. Influence of Scanning Speed on Roughness and Color Contrast

The influence of scanning speed *v* on surface roughness *R_a_* and color contrast ΔE* during nanosecond fiber laser marking of AISI 304 stainless steel are analyzed. The analysis is carried out for single-, double-, and triple-pass scanning marking, followed by a comparative discussion. The scanning speed *v* was varied within the interval from 25 mm/s to 125 mm/s, while three representative raster steps Δ*x* were selected: 20 μm, 50 μm, and 80 μm. The initial surface roughness *R_a_* of the untreated material was *R_a_* = 0.197 μm, and all remaining laser parameters were kept constant (Table 5).

#### 3.2.1. Single-Pass Marking (N = 1) for Roughness

**Figure 11:**
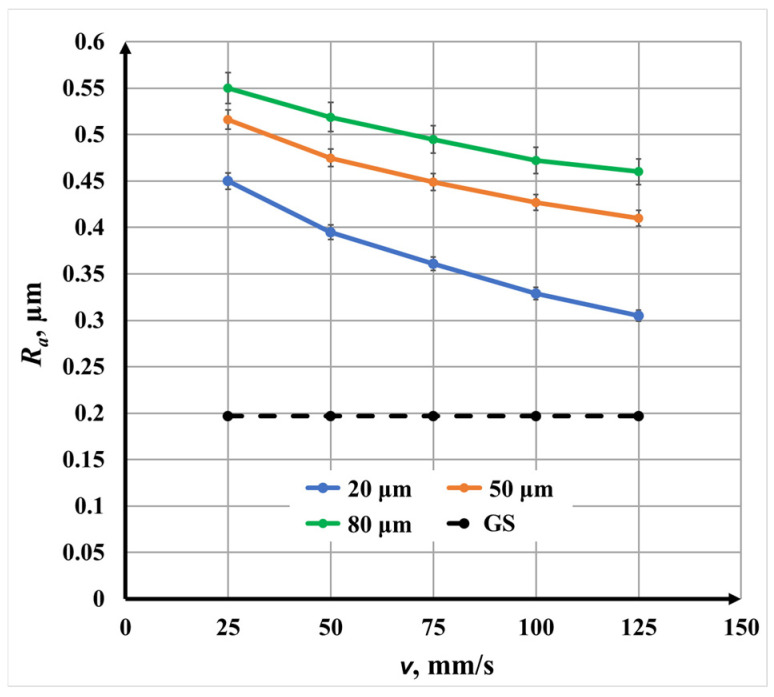
Graphics of the dependence of roughness *R_a_* on the speed for single-repetition three raster steps Δ*x*: blue color—20 μm; orange color—50 μm; and green color—80 μm. The black dotted line is the roughness of the unprocessed surface.

Figure 11 presents the dependence of surface roughness *R_a_* on scanning speed *v* for single-pass laser marking at the three investigated raster steps. For all raster steps, a nonlinear decrease in surface roughness *R_a_* with increasing scanning speed *v* is observed, indicating that speed is a key parameter controlling thermal input and surface modification mechanisms.

At a fixed raster step Δ*x*, increasing the scanning speed *v* reduces the interaction time between the laser beam and the material, leading to lower energy deposition per unit area. Consequently, surface melting and resolidification effects are reduced, resulting in smoother surfaces.

The quantitative change in roughness is as follows:

- For Δ*x* = 20 μm, *R_a_* decreases from 0.45 μm to 0.305 μm;
- For Δ*x* = 50 μm, *R_a_* decreases from 0.516 μm to 0.41 μm;
- For Δ*x* = 80 μm, *R_a_* decreases from 0.55 μm to 0.46 μm as speed *v* increases from 25 mm/s to 125 mm/s.

The rate of roughness *R_a_* reduction decreases with increasing raster step Δ*x*. This indicates that at larger raster steps Δ*x*—where the scanning overlap is lower—the influence of speed on roughness *R_a_* becomes less pronounced. Specifically, the roughness *R_a_* reduction rates are as follows:

- 1.45 × 10^−3^ μm/(mm/s) for Δ*x* = 20 μm;
- 1.06 × 10^−3^ μm/(mm/s) for Δ*x* = 50 μm;
- 0.90 × 10^−3^ μm/(mm/s) for Δ*x* = 80 μm.

This behavior highlights the combined influence of speed *v* and overlap on energy accumulation and surface morphology evolution.

#### 3.2.2. Influence of Speed on Color Contrast for Single-Pass Marking

**Figure 12:**
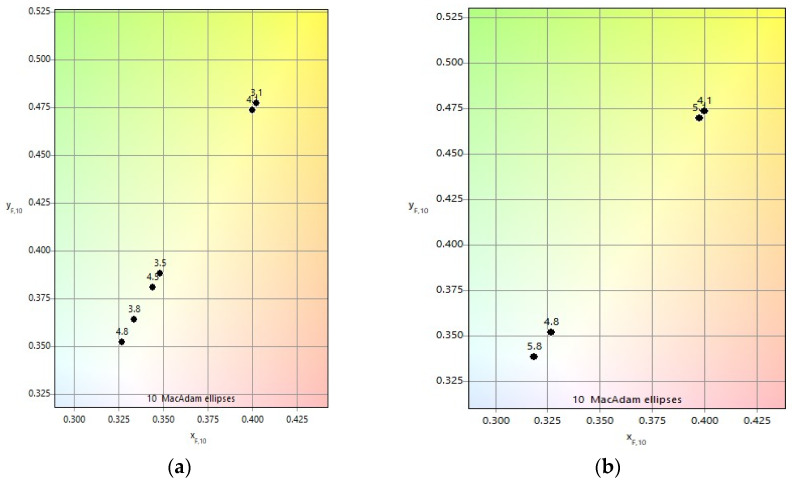
Color diagram for comparing color differences ΔE* and chromaticity distances *ΔH* for speeds *v*: (**a**) 75 mm/s and 100 mm/s and (**b**) 100 mm/s and 125 mm/s.

The influence of scanning speed *v* on color characteristics is illustrated in Figure 12, while quantitative color differences ΔE* and chromatic distances are summarized in Table 7. Comparisons were performed between color pairs obtained at *v* = 75 mm/s and *v* = 100 mm/s (Figure 12a) and between *v* = 100 mm/s and *v* = 125 mm/s (Figure 12b).

For both speed intervals, a clear trend is observed: increasing raster step leads to larger color differences and chromatic distances between the corresponding marked areas. This effect is attributed to reduced overlap and thinner oxide layers at larger raster steps, which enhance sensitivity to changes in scanning speed *v*.

Furthermore, comparisons between higher scanning speeds *v* (100 mm/s and 125 mm/s) reveal larger color differences than those observed at lower speeds *v* (75 mm/s and 100 mm/s). This indicates that at higher speeds, even small variations in absorbed energy significantly affect oxide layer thickness and optical interference conditions.

#### 3.2.3. Double-Pass Marking (N = 2, 0°/90°) for Roughness

**Figure 13:**
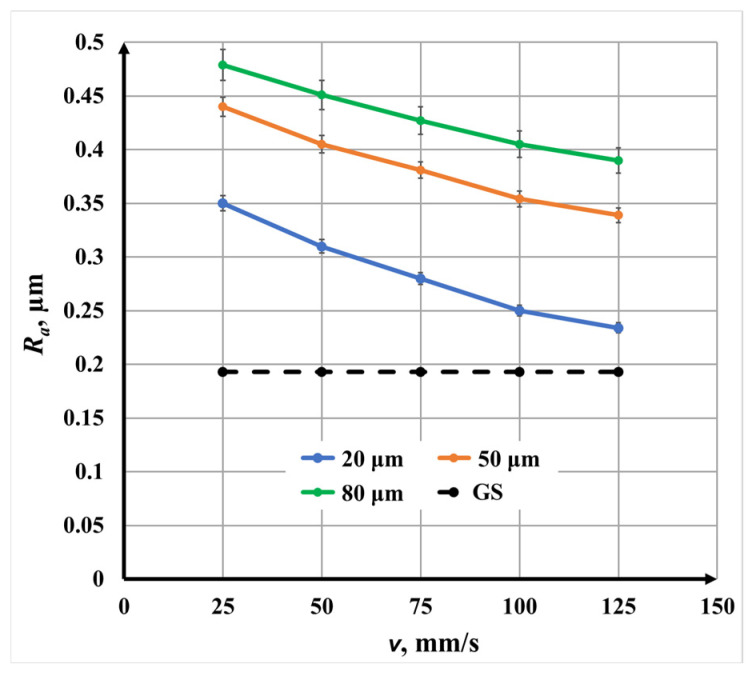
Graphics of the dependence of roughness *R_a_* on the speed *v* for three raster steps *v*: blue color—20 μm; orange color—50 μm; and green color—80 μm. The black dotted line is the roughness of the unprocessed surface.

Figure 13 presents the roughness *R_a_* dependence on scanning speed *v* for double-pass marking. As in the single-pass case, a nonlinear decrease in roughness with increasing speed is observed for all raster steps Δ*x*. However, the absolute roughness *R_a_* values are consistently lower than those obtained with single scanning marking.

The roughness variation is as follows:

- For Δ*x* = 20 μm, *R_a_* decreases from 0.35 μm to 0.23 μm;
- For Δ*x* = 50 μm, *R_a_* decreases from 0.44 μm to 0.34 μm;
- For Δ*x* = 80 μm, *R_a_* decreases from 0.48 μm to 0.39 μm.

The reduced roughness *R_a_* is attributed to the cross-scanning strategy, which redistributes molten material and mitigates the formation of directional grooves. Although speed remains a dominant factor, its effect is partially moderated by the additional pass.

#### 3.2.4. Triple-Pass Marking (N = 3, Angle: 0°/60°/120°) for Roughness

**Figure 14:**
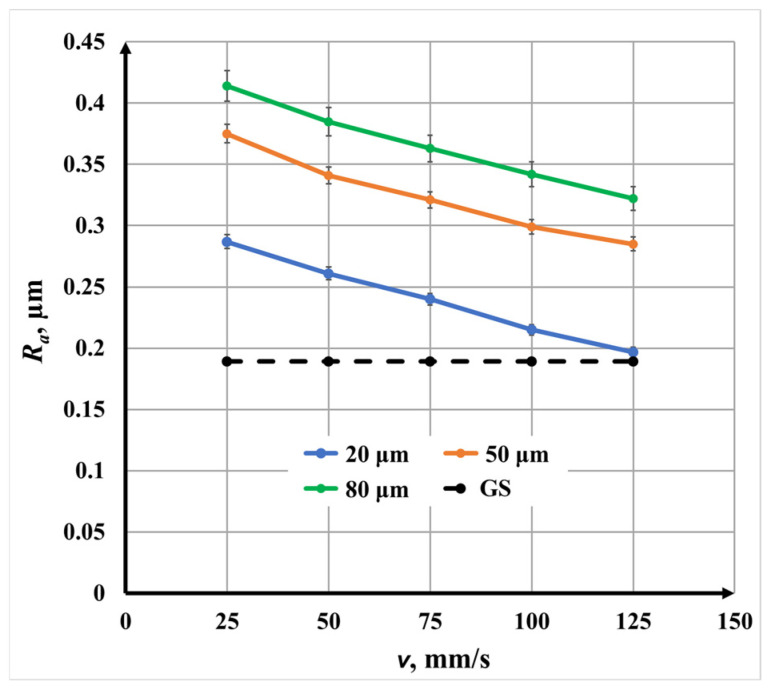
Graphics of the dependence of roughness on the speed for triple-repetition three raster steps Δ*x*: blue color—20 μm; orange color—50 μm; and green color—80 μm. The black dotted line is the roughness of the unprocessed surface.

Figure 14 illustrates the influence of speed *v* on surface roughness for triple-pass marking. The same decreasing trend with increasing speed *v* is observed, but triple-pass marking produces the lowest roughness *R_a_* values across the entire speed range.

Measured roughness *R_a_* values indicate the following:

- From 0.29 μm to 0.20 μm for Δ*x* = 20 μm;
- From 0.375 μm to 0.285 μm for Δ*x* = 50 μm;
- From 0.41 μm to 0.32 μm for Δ*x* = 80 μm.

The quasi-isotropic scanning strategy promotes uniform energy distribution and controlled oxide growth, reducing excessive surface relief even at lower scanning speeds. As a result, the influence of speed on roughness becomes more predictable and stable.

#### 3.2.5. Comparative Analysis of Raster Pass Strategies

**Figure 15:**
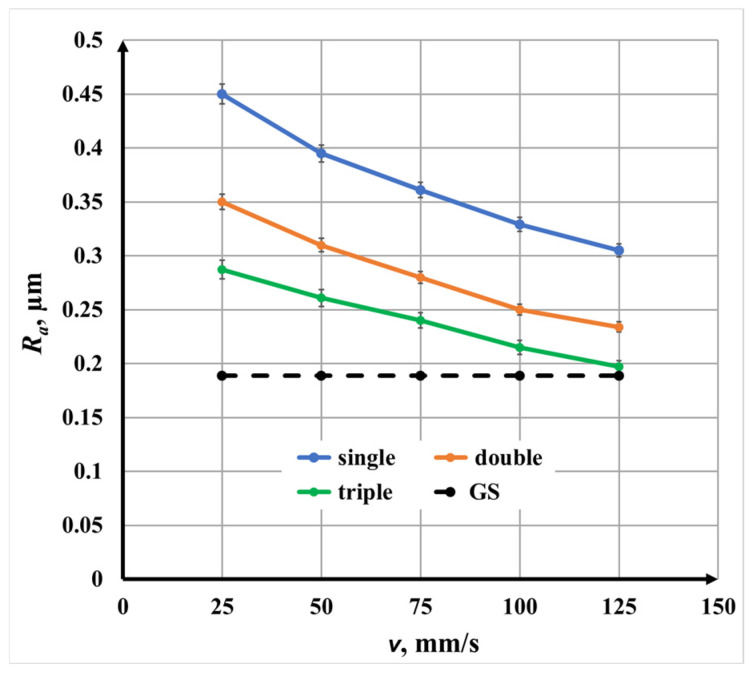
Graphics of the dependence of roughness on the speed *v* for raster step Δ*x* = 80 μm: blue color—single-pass marking; orange color—double-pass marking; and green color—triple-pass marking. The black dotted line is the roughness *R_a_* of the unprocessed surface.

Figure 15 compares the influence of scanning speed *v* on roughness *R_a_* for single-, double-, and triple-pass marking at a fixed raster step Δ*x*. For all marking strategies, increasing speed leads to a nonlinear decrease in roughness *R_a_*. However, the magnitude of roughness strongly depends on the number of repetitions.

At identical speeds,

- Single-pass marking produces the highest roughness *R_a_*;
- Double-pass marking reduces roughness *R_a_* by approximately 30%;
- Triple-pass marking reduces roughness *R_a_* by approximately 65% relative to single marking.

This comparison demonstrates that while scanning speed controls the overall thermal input, the scanning strategy governs the redistribution of energy and material, making repetition number a critical parameter for surface quality optimization.

The summary results demonstrate that scanning speed significantly affects both surface roughness *R_a_* and color contrast. Higher speeds *v* reduce roughness *R_a_* by limiting thermal accumulation, while their influence on color becomes more pronounced at larger raster steps and lower overlap coefficients. Multi-pass scanning not only suppresses excessive roughness *R_a_* but also enhances color saturation by promoting thicker and more uniform oxide layers, particularly in triple repetition configurations.

### 3.3. Influence of Raster Step on Color Difference

This part examines the influence of raster step on the color difference ΔE* obtained by nanosecond laser marking of AISI 304 stainless steel. The color difference ΔE* was calculated relative to the untreated surface, serving as a reference state. The raster step Δ*x* was varied from 20 μm to 80 μm, while five scanning speeds *v* (25 mm/s, 50 mm/s, 75 mm/s, 100 mm/s, and 125 mm/s) were investigated for single-, double-, and triple-pass scanning marking.

Raster step Δ*x* directly controls the degree of overlap between adjacent scan lines, thereby regulating energy density, oxide layer continuity, and interference conditions responsible for color formation.

#### 3.3.1. Single-Pass Marking (N = 1) for Color Difference

**Figure 16:**
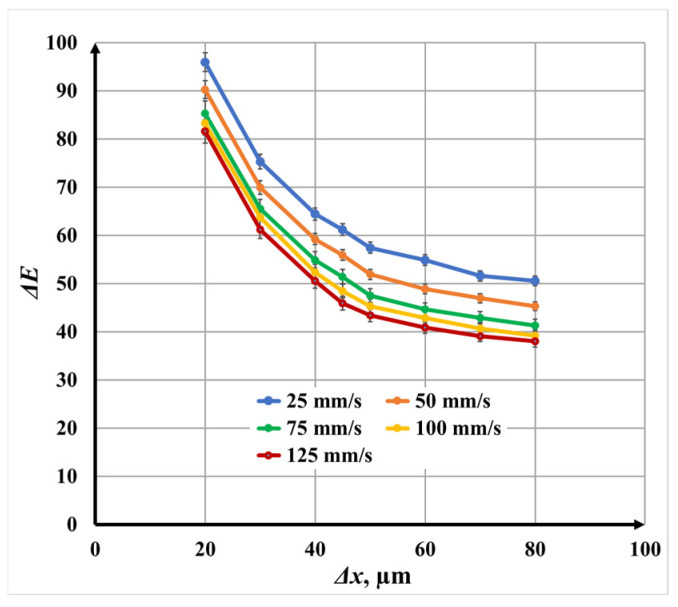
Graphics of the dependence of color difference on the raster step for single repetition for speeds *v*: blue color—25 mm/s; orange color—50 mm/s double; green color—75 mm/s; yellow color—100 mm/s; and red color—125 mm/s.

Figure 16 illustrates the dependence of color difference ΔE* on raster step for single-pass laser marking. For all investigated scanning speeds *v*, a nonlinear decrease in color difference ΔE* with increasing raster step Δ*x* is clearly observed.

The most pronounced reduction in color difference ΔE* occurs in the raster step Δ*x* interval 20 μm to 45 μm, where the curves exhibit steep slopes. In this regime, strong overlap between adjacent scan lines leads to higher cumulative energy deposition, thicker oxide layers, and more intense optical interference effects. When the raster step Δ*x* exceeds 45 μm, the curves become significantly flatter, indicating a transition to a regime dominated by reduced overlap and discontinuous oxide growth.

Quantitatively, at *v* = 25 mm/s, the color difference ΔE* decreases from 95.97 to 50.55, while at *v* = 125 mm/s, it decreases from 81.61 to 38.01 as the raster step Δ*x* increases from 20 μm to 80 μm. Intermediate speeds *v* follow the same trend, confirming that raster step Δ*x* is a dominant factor governing color contrast.

At a fixed raster step Δ*x*, increasing the scanning speed *v* consistently results in lower color differences ΔE*. Compared with *v* = 25 mm/s, the color difference ΔE* decreases by approximately 6.3% at *v* = 50 mm/s, 12.5% at *v* = 75 mm/s, 15.1% at *v* = 100 mm/s, and 17.6% at *v* = 125 mm/s. This behavior reflects the reduced thermal input and thinner oxide layers formed at higher speeds.

#### 3.3.2. Double-Pass Marking (N = 2, Angle: 0°/90°) for Color Difference

**Figure 17:**
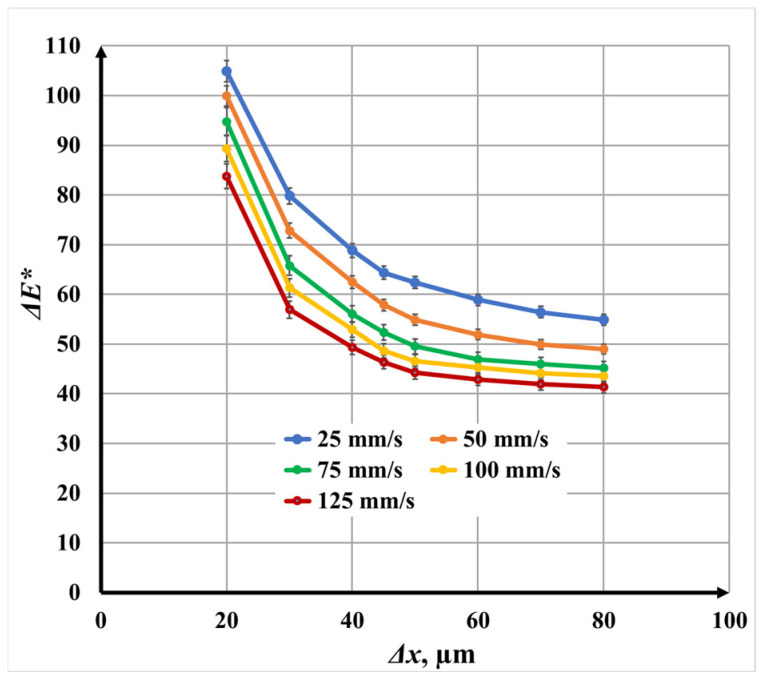
Graphics of the dependence of color difference ΔE* on the raster step for double-pass marking for speeds *v*: blue color—25 mm/s; orange color—50 mm/s double; green color—75 mm/s; yellow color—100 mm/s; and red color—125 mm/s.

The dependence of color difference ΔE* on raster step Δ*x* for double-pass marking is presented in Figure 17. Similar to the single-pass case, color differences ΔE* decrease nonlinearly with increasing raster step Δ*x* for all scanning speeds. However, the absolute values of color difference are systematically higher compared with single repetition.

At *v* = 25 mm/s, the color difference decreases from 104.92 to 54.93, while at *v* = 125 mm/s, it decreases from 83.76 to 41.35 across the raster step Δ*x* range. The steeper slopes observed in the 20 μm to 45 μm interval again indicate strong overlap effects, whereas the flatter behavior beyond 45 μm suggests diminished energy accumulation.

The influence of scanning speed *v* follows the same trend as in single marking: increasing speed leads to lower color differences ΔE*. However, the relative decrease with speed *v* is more pronounced at double repetition, reaching 25.3% when comparing *v* = 25 mm/s and *v* = 125 mm/s. This indicates that the cross-scanning strategy enhances sensitivity to speed variations by redistributing thermal energy over the surface.

#### 3.3.3. Triple-Pass Marking (N = 3, Angle: 0°/60°/120°) for Color Difference

**Figure 18:**
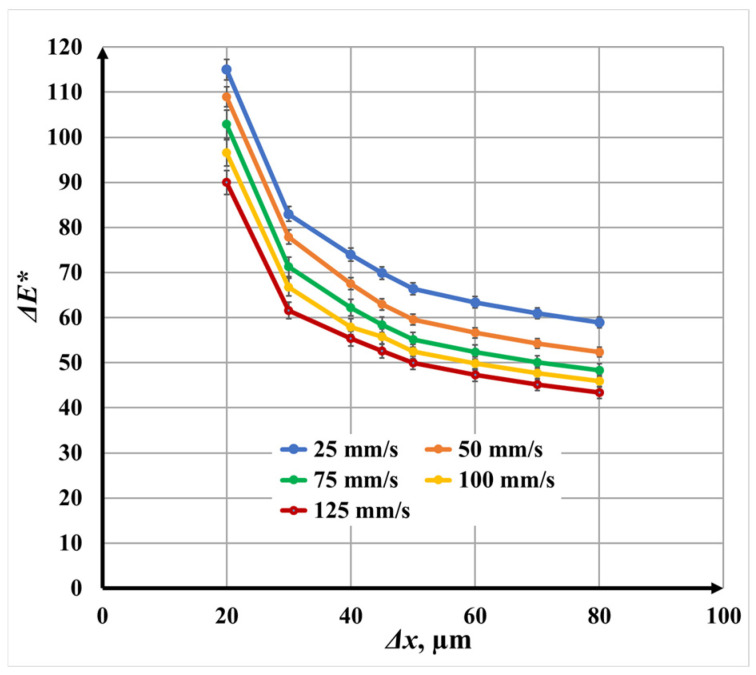
Graphics of the dependence of color difference ΔE* on the raster step for triple repetition for speeds *v*: blue color—25 mm/s; orange color—50 mm/s double; green color—75 mm/s; yellow color—100 mm/s; and red color—125 mm/s.

Figure 18 presents the results for triple-pass marking. For all scanning speeds, triple repetition yields the highest color differences across the entire raster step range.

At *v* = 25 mm/s, color differences ΔE* decreases from 114.99 to 58.95, while at *v* = 125 mm/s, it decreases from 89.96 to 43.42 as the raster step increases from 20 μm to 80 μm. The overall trends mirror those observed for single- and double-pass marking, with a steep decrease at small raster steps and a more gradual decline at larger raster steps.

The higher color differences ΔE* obtained with triple-pass marking are attributed to the quasi-isotropic scanning strategy, which promotes more uniform energy distribution, thicker oxide layer formation, and enhanced optical interference effects. The effect of scanning speed *v* is also amplified, with color differences ΔE* at *v* = 25 mm/s being approximately 27.8% greater than those at *v* = 125 mm/s.

#### 3.3.4. Comparative Analysis at Constant Speed *v* = 25 mm/s

**Figure 19:**
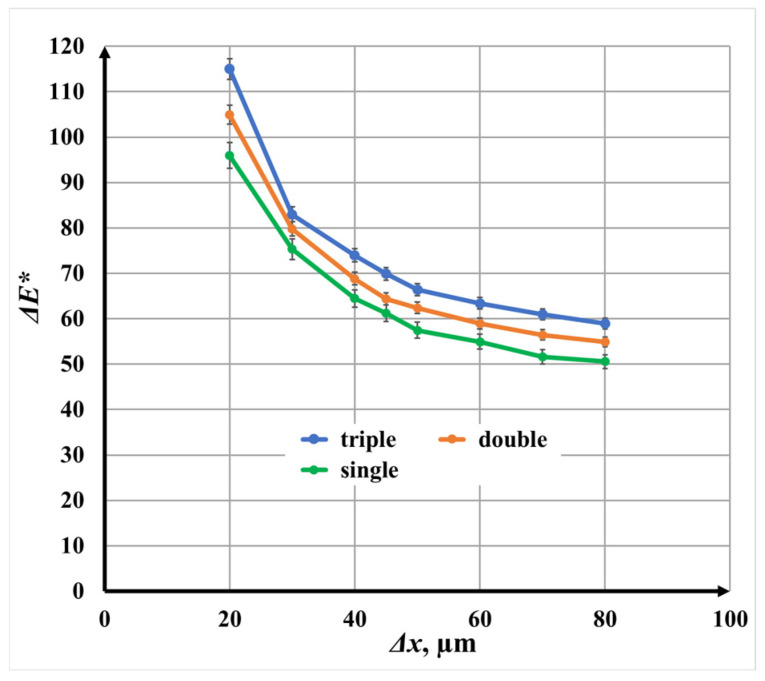
Graphics of the dependence of color difference on the raster step Δ*x* for speeds *v* = 25 mm/s: green color—single repetition; orange color—double repetition; and blue color—triple-pass marking.

Figure 19 compares the influence of raster step Δ*x* on color difference ΔE* for single-, double-, and triple-pass marking at a fixed scanning speed of 25 mm/s. The comparison clearly demonstrates that color difference increases with the number of repetitions across the entire raster step Δ*x* range.

At identical raster steps Δ*x*,

- Double-pass marking produces color differences approximately 9.6% higher than single marking;
- Triple-pass marking increases color difference by approximately 19.8% relative to single marking.

These results confirm that color difference ΔE* is strongly governed by the surface morphology and oxide layer thickness, both of which are enhanced by multiple scanning passes. Increased repetition leads to higher cumulative energy input, improved oxide continuity, and more pronounced interference coloration.

The summary results demonstrate that raster step Δ*x* is a critical parameter controlling color contrast in laser marking of AISI 304 stainless steel. Small raster steps Δ*x* ≤ 45 μm promote strong overlap and high color differences ΔE*, while larger raster steps Δ*x* lead to diminished overlap and reduced optical contrast. Increasing scanning speed *v* consistently lowers color difference ΔE*, whereas increasing the number of scanning pass repetitions significantly enhances it. Among all investigated parameters, triple-pass marking combined with small raster steps Δ*x* and low scanning speeds *v* yields the highest color contrast, making this configuration optimal for high-visibility and anti-counterfeiting laser markings.

## 4. Conclusions

This study shows that the morphology obtained during raster laser marking of AISI 304 stainless steel plays a crucial role in the resulting surface texture and color differences ΔE*. By applying single-, double-, and triple-pass marking strategies with different angular orientations, different surface structures were generated, each of which showed a different behavior toward the raster step Δ*x* and scanning speed *v*. The influence of the scanning overlap coefficient *k_soc_* on the resulting color gamut was also indirectly established. The results show clear correlations between the processing parameters on one hand and the surface roughness *R_a_*, RGB colors, color differences ΔE*, and chromatic distances Δ*H* on the other hand.

The following results were achieved:

- A graphical dependence of the roughness *R_a_* on the raster step Δ*x* and speed *v* for single, double and triple marking was obtained;
- A trend of color change and color saturation with changing the raster step Δ*x* and speed *v* for the three types of markings studied was obtained;
- The color differences ΔE* were determined for five different speeds *v* in the investigated raster step Δ*x* interval.

Overall, the achieved results provide a deeper understanding of how controlled morphological modification affects color in laser marking of stainless steel. Similar results have been achieved by other researchers [21,22].

## Figures and Tables

**Table 1:** Chemical composition of AISI 304 stainless steel [20].

Chemical Element	Content, %	Chemical Element	Content, %
C	0.075	Si	0.80
Cr	17.7	Cu	0.30
Ni	10.2	P	0.08
Mn	2.0	S	0.02
Ti	0.50	Fe	Balance

**Table 2:** Basic physical properties of AISI 304 stainless steel [20].

Parameter	Value
Coefficients of thermal conductivity *k*, W/(m·K)	27
Density *ρ*, kg/m^3^	7850
Specific heat capacity *c*, J/(kg·K)	504
Coefficients of thermal diffusivity *a*, m^2^/s	4.3 × 10^−6^

**Table 3:** Parameters of laser systems used in the research [20].

Parameter	Value	Parameter	Value
Wavelength *λ*, nm	1064	Pulse energy *E_p_*, mJ	0.08–1.00
Average power *P*, W	1–20	Pulse power *P_p_*, kW	0.80–10.0
Frequency *ν*, kHz	20–250	Beam quality *M*^2^	<1.1
Pulse duration *τ*, ns	100	Scan speed *v*, mm/s	1–20,000
Positioning accuracy	±2.5 μm	Efficiency, %	40
Diameter in focus *d*, µm	70		

**Table 4:** Specifications of the OLS5100 laser microscope [20].

Parameter	Value	Parameter	Value
Total magnification	54× – 17,280×	Measurement accuracy	±1.5%
Field of view	16 μm to 5120 μm	Laser wavelength	405 nm
Display resolution	1 nm	Laser source power	0.95 mW
Max measuring points	4096 × 4096 pixels		

**Table 5:** Parameters that do not change during experiments [20].

Parameter	Value	Parameter	Value
Power *P*, W	20	Number of repetition *P_p_*, kW	1
Frequency *ν*, kHz	20	Defocusing Δ*f*	0
Pulse duration *τ*, ns	100	Diameter of working spot *d*, µm	70

**Table 6:** Colors obtained during a raster marking of a sample of AISI 304 stainless steel with a fiber laser are indicated in each cell as a background.

**Δ** ***x*** **, μm** ***v*** **, mm/s**	**20**	**30**	**40**	**45**
25	1.1 12/0/136	1.2 15/36/139	1.3 13/135/45	1.4 13/135/53
50	2.1 100/215/50	2.2 153/255/153	2.3 255/255/161	2.4 255/255/170
75	3.1 255/255/90	3.2 255/255/144	3.3 255/255/173	3.4 255/255/183
100	4.1 255/255/96	4.2 255/255/149	4.3 255/255/180	4.4 255/255/192
125	5.1 255/255/102	5.2 255/255/156	5.3 255/255/186	5.4 255/255/200
**Δx, μm** **v** **, mm/s**	**50**	**60**	**70**	**80**
25	1.5 65/140/52	1.6 155/230/150	1.7 255/255/182	1.8 255/255/185
50	2.5 255/255/181	2.6 255/255/190	2.7 255/255/197	2.8 255/255/202
75	3.5 255/255/195	3.6 255/255/205	3.7 255/255/212	3.8 255/255/219
100	4.5 255/255/202	4.6 255/255/212	4.7 255/255/223	4.8 255/255/231
125	5.5 255/255/210	5.6 255/255/221	5.7 255/255/232	5.8 255/255/245

**Table 7:** Comparative results of color differences ΔE* and chromatic distances Δ*H* for different marked areas.

Marked Zone	Raster Step, μm	Speed, mm/s	Color Difference	Chromatic Distance
3.1/4.1	20	75/100	2.28	0.0044
3.5/4.5	50	75/100	3.50	0.0081
3.8/5.8	80	75/100	6.06	0.0140
4.1/5.1	20	100/125	2.37	0.0046
4.8/5.5	80	100/125	7.09	0.1371

## Data Availability

The original contributions presented in this study are included in the article. Further inquiries can be directed to the corresponding author.
